# TNF-**α** Promotes IFN-**γ**-Induced CD40 Expression and Antigen Process in Myb-Transformed Hematological Cells

**DOI:** 10.1100/2012/621969

**Published:** 2012-04-01

**Authors:** Wenyi Gu, Jiezhong Chen, Lei Yang, Kong-Nan Zhao

**Affiliations:** ^1^Australian Institute for Bioengineering and Nanotechnology, The University of Queensland, Brisbane, QLD 4072, Australia; ^2^Illawarra Health and Medical Research Institute, University of Wollongong, NSW 2522, Australia; ^3^School of Medicine and Medical Management, Hangzhou Normal University, Hangzhou 310036, China; ^4^UQ Centre for Clinical Research, The University of Queensland, Herston, Brisbane, QLD 4029, Australia

## Abstract

Tumour necrosis factor-**α**, interferon-**γ** and interleukin-4 are critical cytokines in regulating the immune responses against infections and tumours. In this study, we investigated the effects of three cytokines on CD40 expression in Myb-transformed hematological cells and their regulatory roles in promoting these cells into dendritic cells. We observed that both interleukin-4 and interferon-**γ** increased CD40 expression in these hematological cells in a dose-dependent manner, although the concentration required for interleukin-4 was significantly higher than that for interferon-**γ**. We found that tumour necrosis factor-**α** promoted CD40 expression induced by interferon-**γ**, but not by interleukin-4. Our data showed that tumour necrosis factor-**α** plus interferon-**γ**-treated Myb-transformed hematological cells had the greatest ability to take up and process the model antigen DQ-Ovalbumin. Tumour necrosis factor-**α** also increased the ability of interferon-**γ** to produce the mixed lymphocyte reaction to allogenic T cells. Furthermore, only cotreatment with tumour necrosis factor-**α** and interferon-**γ** induced Myb-transformed hematological cells to express interleukin-6. These results suggest that tumour necrosis factor-**α** plays a key regulatory role in the development of dendritic cells from hematological progenitor cells induced by interferon-**γ**.

## 1. Introduction

Cytokines are key components of the immune system and play a crucial role in immune cell developments. An easy model to study their biological functions is to use hematological progenitor cells. Myb-transformed hematological cells (MTHCs) were originally derived from fetal mouse liver and were immortalized by retrovirally vectored *Myb* gene transformation [1–3]. Since then, the cell line has been characterized and used as a model of hematological stem cells to investigate the development of hematological progenitor cells to dendritic cells (DCs) and macrophages [1–3]. Previous studies have shown that cytokines play key regulatory roles in the development of MTHCs into either DCs or macrophages [1–3]. Particularly, IL-4 and IFN-*γ* were the critical cytokines that promote the differentiation of the progenitor cells into either DC or macrophage lineages. For example, IL-4 plus TNF-*α* was shown to promote the MTHCs to develop into macrophages, while cotreatment of IFN-*γ* and TNF-*α* produced DCs [1, 4–6]. 

TNF-*α* was identified as an endotoxin-induced factor that caused the necrosis of certain murine tumours *in vivo*  [7]. As a powerful immune modulator, TNF-*α* is involved in systemic inflammation and is a member of the cytokines that stimulate the acute-phase reaction. TNF-*α* also plays an important role in the development and maintenance of DCs [8, 9]. It promotes expression of MHC class II and costimulatory molecules (CD80 and CD86) [10]. In addition, TNF-*α* is essential for host defenses against mycobacteria and other granulomatous pathogens [11]. Anti-TNF-*α* treatment of DCs has been shown to cause apoptosis, indicating the important role of TNF-*α* in DC survival [11]. In DC culture, addition of TNF-*α* into the culture medium without plasma could maintain DC viability, while addition of GM-CSF or IL-4 to the culture medium had no such an effect [12]. 

DCs are professional antigen-presenting cells (APCs) involved in both innate and adaptive immune responses [4, 13]. DCs also play a key role in the induction of immune tolerance [14, 15]. Upon stimulation, they secrete cytokines and activate T cells. The functions of DCs are closely related to their maturation status and expression of cell markers for immune modulators such as costimulation factors, CD40 and B7-1 on the cell surface [16]. Interaction between CD40 and CD40L is considered to be very important in DC maturation and antigen presentation of DCs to T cells [17, 18]. Other studies showed that CD40 was important for the Th1 response, but not for the Th2 response [19]. Understanding of the factors that contribute to the differentiation and maturation of DCs and their relationship to the function is necessary for possible manipulations of DCs for future treatments of immune diseases or cancers [20–22]. 

Although the role of IL-4 and IFN-*γ* in the development of hematological progenitor cells into DCs or macrophages has been well documented, the biological function of TNF-*α* to modulate IL-4 and IFN-*γ* levels to induce the development and differentiation of DCs from progenitor cells is less well studied. This prompted us to evaluate the regulatory role of TNF-*α* in the induction of dendritic cells from MTHCs with these two key cytokines. We found that TNF-*α* promoted the IFN-*γ*-induced development of DCs and the generation of functional DCs from MTHCs through CD40 expression and antigen processing. Cotreatment of TNF-*α* and IFN-*γ* induced MTHCs to express IL-6 mRNA, and TNF-*α* reduced the production of IL-12 induced by IFN-*γ*. These findings are valuable for understanding the role of TNF-*α* in DC cell development and immunological function.

## 2. Material and Methods 

### 2.1. Cell Lines, Cytokines, and Cell Treatment

The MTHCs used in this study were prepared from C3H mouse using the same method as described previously [23]. The MTHCs were maintained in modified IMEM medium (Invitrogen) containing 50 unit/mL of GM-CSF and 10% fetal calf serum (FCS). Mouse GM-CSF, IL-4, and IFN-*γ* were produced in insect Sf9 cells by infecting the cells with baculovirus expressing the corresponding genes. The concentrations were titered as described previously [23]. Mouse TNF-*α* was purchased from Sigma and was stored at −80°C in small aliquots until use. 

For cytokine treatment, the cells were cultured in 24- or 96-well plates and the cytokines were diluted in culture medium and added directly to the cells. For dose-dependent CD40 expression experiments, the cells were seeded at a density of 1 × 10^6^ cells/well in a 24-well plate and the three corresponding cytokines were added, respectively. The concentrations for IL-4 ranged from 25 to 800 units/mL for IFN-*γ* they were from 0.5 to 16 units/mL and for TNF-*α* from 50 to 1600 units/mL in 2-fold serial dilutions. Twenty-four or 48 hours after treatment the cells were collected for CD40 expression assays using FACS. For co-treatment with TNF-*α* + IFN-*γ* or TNF-*α* + IL-4, 200 units/mL of TNF-*α* was used unless it was specifically indicated. 

### 2.2. FACS Analysis for CD40 Expression

The cells treated with different cytokines at various doses were harvested by centrifugation and washed once with 2% fetal calf serum (FCS)/PBS. The cells were then incubated with 1 : 10 diluted monoclonal antibody to mouse CD40 produced from FGK4.5 hybridoma for 1 hour at 37°C. The cells were washed twice with 2% FCS/PBS, followed by incubation with the secondary antibody anti-mouse IgG2 conjugated with FITC (Santa Cruz Biotechnology) for 1 hour at room temperature. The cells were washed three times using 2% FCS/PBS, and fixed in 2% paraffin-formaldehyde (PFA)/PBS for flow cytometry (FACS) analysis.

### 2.3. Antigen Processing

DQ-ovalbumin (DQ-OVA) (Invitrogen) is a self-quenched conjugate of ovalbumin that exhibits green fluorescence when proteolytic degradation in the cells, which can be measured by flow cytometry. DQ-OVA was added into 100 *μ*L of suspension cells at a concentration of 10 *μ*g/mL. The cells were incubated at 37°C for 1 hour. They were then washed with 2% FCS/PBS twice, fixed in 2% PFA/PBS and analyzed using both flow cytometry and confocal microscopy. 

### 2.4. Mixed Lymphocyte Reaction

The mixed lymphocyte reaction (MLR) analysis was carried out as previously described [23]. Briefly, the responder cells were C57BL/6 mouse spleen cells passed twice through nylon wool column, and the stimulators were MTHCs treated with cytokines and irradiated by a ^137^Cs irradiator (IBL437C, Australia). The cells were plated in 96-well plates at various ratios of responder verse stimulator cells, and at least 3 repeats for each ratio were set up in the plates. After 4-5 days, the T-cell proliferation was measured by the uptake of [3H]-thymidine (1 pCil/well, 6.7CilmM; ICN, Costa Mesa, Calif, USA), which was added during the final 18 hours of the culture. Cells were harvested onto glass fiber filter paper with an automated 96-well harvester (Wallac, Turku Finland), and [3H]-thymidine incorporation was determined using a liquid scintillation counter.

### 2.5. Preparation of CD40 Ligand (CD40L) from Insect Cells

Insect Sf9 cells were cultured in T-75 or T-125 flasks with IPL-41 medium (Invitrogen) for 3-4 days at 27°C. When the cells were 80% confluent, they were infected with baculovirus that expresses mouse CD40L. After culturing for 4-5 days, the baculovirus-infected cells were harvested and pelleted by centrifugation. The cell pellets were resuspended in homogenization buffer and sonicated for 20 seconds on ice. A volume of 6.6 mL homogenizer was transferred into each 10 mL ultracentrifuge tubes and underlayered with 2.6 mL 40% sucrose solution. The tubes were centrifuged 96000 ×g for 1 hour at 4°C. The interfaces were recovered and transferred into a sterilized 50 mL tube and then diluted to 20 mL with PBS. Ten mL of this preparation was transferred into a Beckman ultracentrifuge tube, and the tubes were centrifuged using Ti-50 rotor at 36000 rpm for 30 minutes. The supernatant was discarded, and the pellet was resuspended in 10 mL PBS. This centrifugation was repeated once more. The pellet was finally resuspended into 2 mL PBS, and the solution was stored at −70°C in small aliquots. The preparation was analysed by Western blotting to detect the CD40L protein (data not shown) with goat anti-mouse CD40L IgG (Santa Cruz Biocenology).

### 2.6. IL-12p40 Protein Measurement

The purified anti-mouse IL-12-p40 antibody-coated ELISA kit (ELISA capture, BD Bioscience, Australia) was purchased and used to measure IL-12p40 protein in the cultural supernatant of MTHCs treated with different cytokines and CD40L, according to the manufacturer's manual.

### 2.7. RT-PCR

RT-PCR analysis was carried out as previously described [24]. Briefly, the treated and untreated cells were harvested for total RNA extraction using TRIzol reagent (Invitrogen) according to the manufacturer's instruction. Reverse transcription reactions were performed with oligo-dT primer. The primers for IL-6 RT-PCR were as follows: forward: 5′-TGCTGGTGACAACCACGGCC; reverse: 5′-GTACTCCAGAAGACCAGAGG. This resulted in an amplified product size of 308 nucleotides. Mouse *β*-actin was used as internal control (forward: 5′-GCTACAGCTTCACCACCACA; reverse: 5′-TCTCCAGGGAGGAAGAGGAT). The PCR was performed in 20 *μ*L volume with 2.5 *μ*L 1 : 10 diluted reverse transcription product. The PCR program was preheating at 95°C for 5 min; the cycle consisted of 94°C for 45 sec, 56°C for 1 min, and 72°C for 2 min for a total of 35 cycles.

## 3. Results

### 3.1. TNF-*α* Increased CD40 Expression Induced by IFN-*γ*, but Not by IL-4

**Figure 1:**
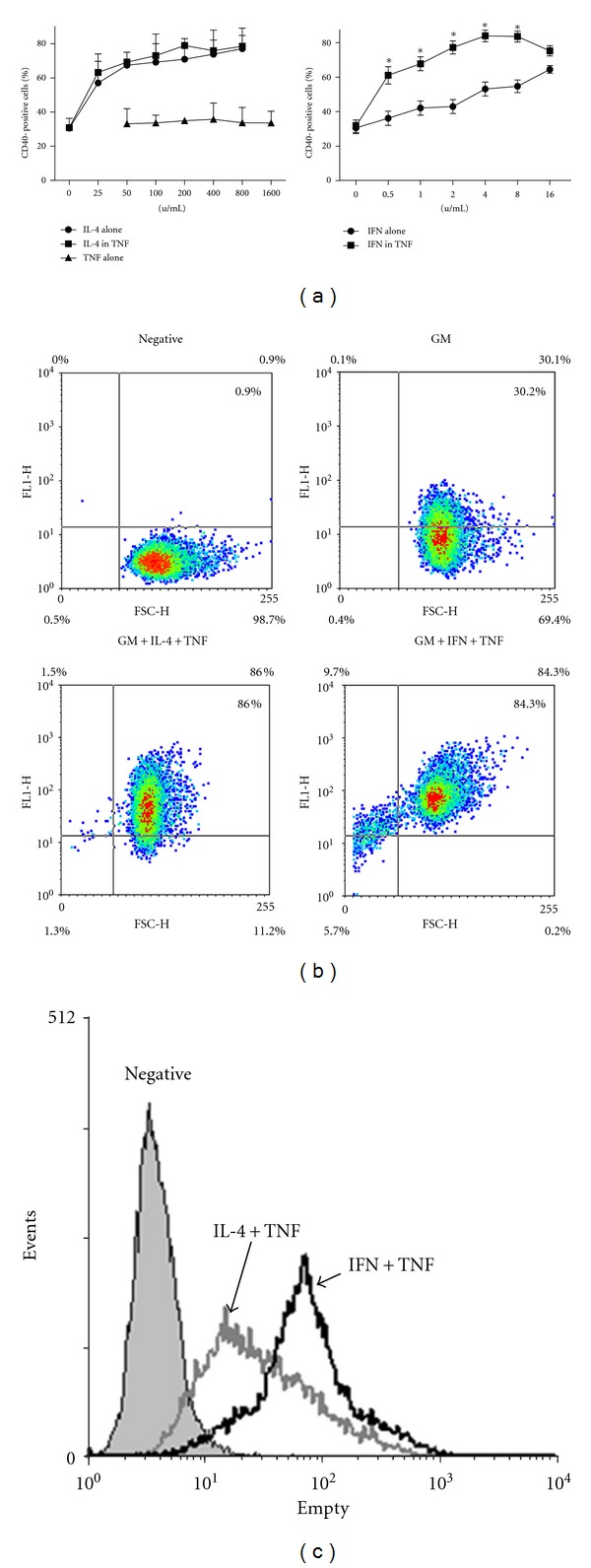
The effect of cytokine treatment on CD40-expression in MTHCs. (a) The FACS results show CD40-positive cells (%) after the 3 cytokine treatments or cotreatments at different doses. The doses for IFN-*γ* (IFN), TNF-*α* (TNF), and IL-4 treatment alone were as labeled. For TNF-*α* co-treatment with IFN-*γ* and IL-4, TNF-*α* dose was 200 units/mL and IFN-*γ* and IL-4 doses were as labeled. The data were the mean ± SE from 3 separate experiments after 48 hours of treatment. (b) The diagrams show the dot plot analysis of the results from above treatments. Negative: the antibody isotype control. GM: GM-CSF alone. (c) shows the FACS histogram analysis of fluorescent intensity for GM + IL-4 + TNF-*α* (IL-4 +TNF) and GM + IFN-*γ* + TNF-*α* (IFN+TNF) groups in (b). Negative: the antibody isotype control.

We firstly examined the effect of individual cytokines on CD40 expression in MTHCs. CD40 expression in MTHCs treated with IL-4 or IFN-*γ* alone was increased in a dose-dependent manner (Figure 1(a)). TNF-*α* alone did not enhance CD40 expression even though it was used in a dose up to 1600 units/mL (Figure 1(a)). To examine whether TNF-*α* could increase CD40 expression induced by either IL-4 or IFN-*γ* in MTHCs, we cotreated the cells with TNF-*α* +IL-4 or TNF-*α* + IFN-*γ*. Co-treatment of TNF-*α* and IL-4 did not further increase CD40 expression of MTHCs, while co-treatment of TNF-*α* and IFN-*γ* significantly increased CD40 expression (Figure 1(a)). 

**Figure 2:**
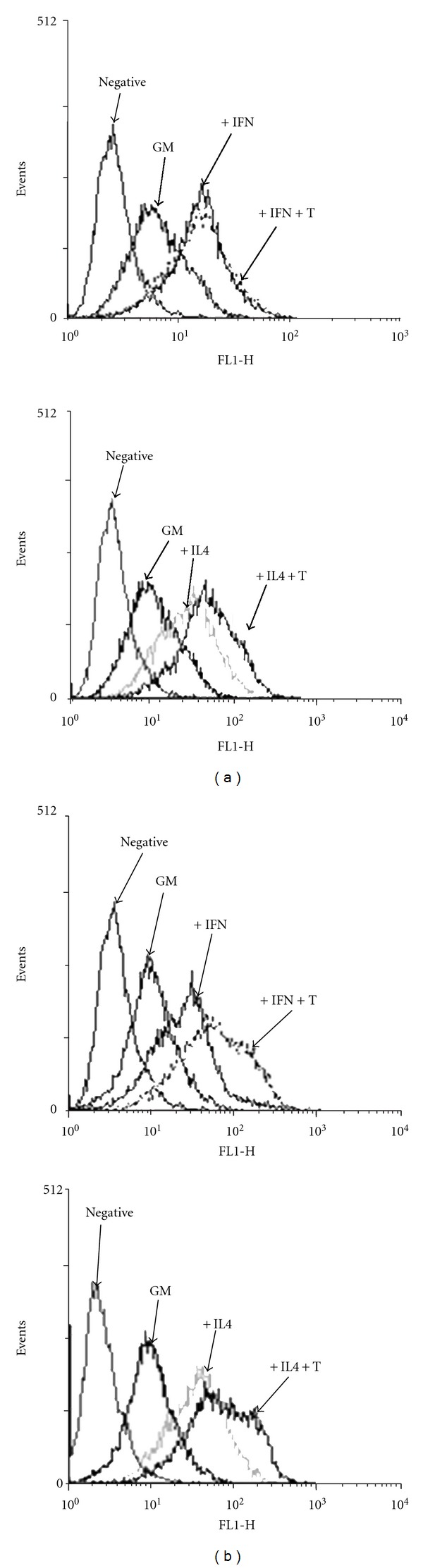
TNF-*α* promotes CD40 expression induced by IFN-*γ* after 48 hours of treatment. The histogram analysis of CD40 expression in MTHCs treated with either IFN-*γ* (2 units/mL) or IFN-*γ* + TNF-*α* (broken line, IFN+T) is the representative results of FACS analysis from two separate experiments at 24 and 48 hours after treatment (upper panel). IL-4 and IL-4 + TNF-*α* (IL-4+T) treatments are shown in the lower panel. Negative and GM controls are the same as in Figure 1.

When using dotplot to analyze the co-treatment results, we found that the CD40 positivity was quite different, even though the final percentages of positive cells were similar (86% and 84.3%, Figure 1(b)). Co-treatment of TNF-*α* and IFN-*γ* resulted in more dead cells (15.4%) than TNF-*α* + IL-4 treatment (2.8%, Figure 1(b)). When these dead cells were excluded, the percentage of positive cells was higher than 86%. This is further supported by the comparison of their mean fluorescence intensity of CD40 expression (68.95 for the TNF-*α* +IFN group versus 34.11 for TNF-*α* +IL-4 group, Figure 1(c)). Besides, comparing with IL-4 + TNF-*α* treatment, we found that the co-treatment of IFN-*γ* and TNF-*α* needed to be at least 48 hours to promote CD40 expression, as 24-hour treatment was not long enough to increase CD40 expression (Figure 2), suggesting that IFN-*γ*-induced CD40 expression enhanced by TNF-*α* was also dependent on the length of the treatment.

### 3.2. Cotreatment of IFN-*γ* and TNF-*α* Promoted Antigen Process

**Figure 3:**
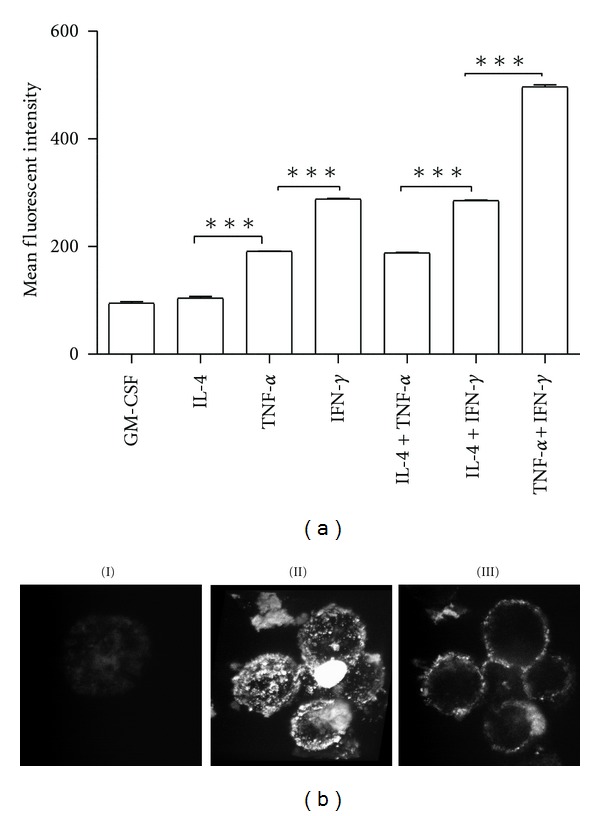
The ability of cytokine-treated MTHCs to process DQ-OVA antigen *in vitro*: (a) the mean fluorescent intensity of FACS analysis shows that MTHCs treated with different cytokines have the different abilities to process DQ-OVA antigen. Data are expressed as the mean ± SE of three independent experiments. ***P < 0.001. (b) The representative images of confocal microscopy show the process of DQ-OVA by MTHCs treated with IFN-*γ* + TNF-*α*: (I) MTHCs cultured with GM-CSF only scarcely show any DQ-OVA fluorescent signal on the cell surface. (II) MTHCs treated with IFN-*γ* + TNF-*α* have strong DQ-OVA signals on the cell surfaces. (III) The Z-stack image of cross-scanning of the positive cells confirms that the processed DQ-OVA antigen (fluorescent signals) was localized on the cell membranes.

We next examined the effect of the treatment of three cytokines on the ability of MTHCs to take up and process antigen DQ-OVA. DQ-OVA has been used as a model for antigen processing and presentation. According to the FACS analysis of DQ-OVA fluorescence, MTHCs grown in GM-CSF medium had a very weak ability to take up and process DQ-OVA (Figure 3(a)). Treatment with IL-4 alone did not increase the antigen uptake and processing ability of MTHCs, whereas the treatment with TNF-*α* or IFN-*γ* could significantly increase this ability of the MTHCs (Figure 3(a)). Co-treatment of IL-4 with either TNF-*α* or IFN-*γ* could further increase the fluorescence intensity (Figure 3(a)), suggesting that IL-4 itself did not have any effect on antigen uptake and processing. In contrast, co-treatment with IFN-*γ* and TNF-*α* had the greatest ability to increase the fluorescence intensity (Figure 3(a)) and show the additive effects on promoting MTHCs to take up and process the antigen. 

To confirm that the antigen was taken up and processed by MTHCs cotreated with IFN-*γ* and TNF-*α*, we examined the distribution of DQ-OVA in the cells using confocal microscopy. The images showed that MTHCs treated with IFN-*γ* plus TNF-*α* exhibited strong fluorescent signals on the cell surfaces (Figure 3(b), II), comparing with GM-CSF (Figure 3(b), I). The cross-cell images demonstrated that the fluorescent signals were localized on the cell surfaces (Figure 3(b), III). These data suggest that DQ-OVA antigen was processed and presented on the cell membrane. 

### 3.3. TNF-*α* Increased IFN-*γ* to Produce Strong Mixed Lymphocyte Reaction Response to Allogenic T Cells

**Figure 4:**
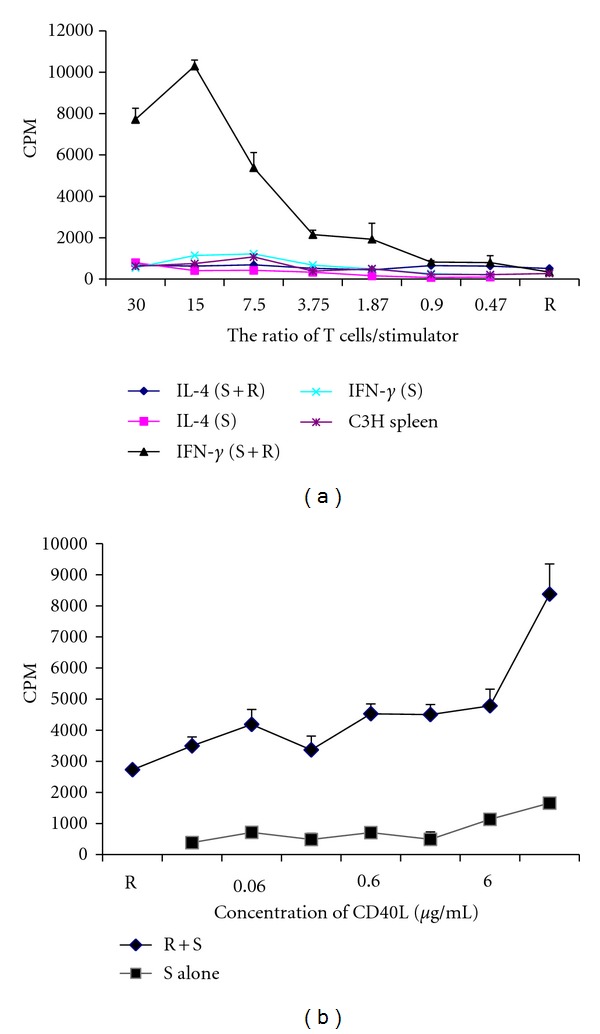
The ability of cytokine-treated MTHCs to stimulate mixed lymphocyte reaction (MLR). (a) The MLR results with MTHCs cotreated with 200 unit/mL TNF-*α* and IL-4 or IFN-*γ* as stimulator (*S*) and enriched T cells from C57BL/6 mouse spleen as responders (*R*). Stimulators and responders were set up at different ratios as indicated. (b) The effect of CD40L treatment on MLR: different doses of CD40L were added to MLR with IFN-*γ* + TNF-*α*-treated MHTCs as stimulators and enriched C57BL/6 spleen T cells as responders (at the ratio of 3.75). The data were the mean ± SE of three separate experiments.

To examine whether TNF-*α* could increase IL-4 or IFN-*γ* to stimulate T-cell response in MLR, we used T cells taken from a spleen cell population of C3H3 mice as the responder and MTHCs treated with 50 units/mL TNF-*α* together with either IL-4 or IFN-*γ* as the stimulator. The stimulator was irradiated before adding in MLR. The results showed that only the cells treated with TNF-*α* together with IFN-*γ* could stimulate a strong MLR response to allogenic T cells (Figure 4(a)), suggesting again that TNF-*α* could promote IFN-*γ* but not IL-4 in stimulating T cells. Furthermore, the cell ratio of responder/stimulator played a key role in MLR response to allogenic T cells, with a ratio of 15 producing the maximum reaction (Figure 4(a)). 

A previous study has shown that DCs cultured with CD40L increased capacity to stimulate allogeneic T cells [25]. To investigate if stimulation of MLR by TNF-*α* + IFN-*γ*-treated MTHCs was associated with CD40 expression or activation, we also used CD40L in MLR. We observed that CD40L significantly increased the ability of MTHCs treated with TNF-*α* and IFN-*γ* to stimulate MLR response in a dose-dependent manner (Figure 4(b)).

### 3.4. Cotreatment of TNF-*α* and IFN-*γ* Induced IL-6 mRNA Expression in MTHCs

**Figure 5:**
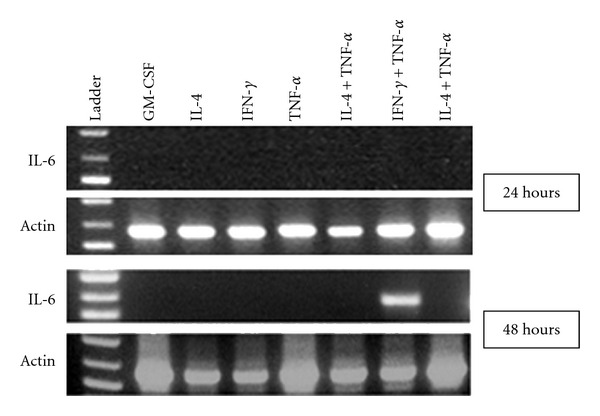
Expression of IL-6 mRNA in cytokine-treated MTHCs: the RT-PCR results show that an IL-6 band is present only in the MTHCs treated with IFN-*γ* plus TNF-*α* at 48 hours but not in other treatments. Ladder: DNA ladders.

To investigate whether co-treatment of TNF-*α* and IFN-*γ* could induce expression of cytokines that have been shown to be involved in the CD40 expression and antigen processing, we used RT-PCR analysis to examine expression of six cytokines including IL-1*α* and *β*, IL-3, IL-5, IL-6, and IL-7 in the MTHCs. The MTHCs were treated with the three cytokines individually (IL-4; IFN-*γ* and TNF-*α*) or with a combination of two out of the three cytokines for 24 or 48 h. Except for IL-6, the MTHCs did not express any mRNAs of the other five cytokines examined (data not shown). IL-6 mRNA was detected in the cells treated with a combination of TNF-*α* and IFN-*γ* at 48 hours after treatment but not at 24 hours (Figure 5). The data suggests that TNF-*α* plus IFN-*γ* treatment to MTHCs can induce IL-6 mRNA expression in these cells, which is similar to CD40 expression.

### 3.5. Cotreatment with TNF-*α* and IFN-*γ* Did Not Promote IL-12 Production in MTHCs

IL-12 is naturally produced by DCs and facilitate antigen presentation [26]. To further investigate the possibility of IL-12 involvement in increased antigen process by the treatment of TNF-*α* and IFN-*γ*, we examined IL-12 protein in the MTHC culture medium using ELISA (Table 1). CD40L was added to stimulate the production. As shown in Table 1, without presence of CD40L, IL-12 was scarcely detected in the medium. IL-12 was detected in the presence of CD40L, with a high level detected in the medium from the IFN-*γ*-treated MTHC culture (Table 1). But the level was reduced when treated with a combination of IFN-*γ* and TNF-*α* (Table 1), indicating that TNF-*α* decreased IFN-*γ*-induced IL-12 secretion in MTHCs.

## 4. Discussion

The process of progenitor cells differentiating into macrophages and DCs is critical for both innate and adaptive immunity. CD40 expression is an essential marker for the progenitor cells to develop into mature antigen-presenting cells [27–29]. CD40 is also a co-stimulatory molecule, and its ligation with the ligand on T cells can induce the T-cell activation and production of different proinflammatory cytokines. Therefore, in this study, we investigated the effects of treatments with the three important cytokines on CD40 expression and antigen process in MTHCs, a model of hematological progenitor cells. Both IL-4 and IFN-*γ* can induce CD40 expression in a dose-dependent manner, and TNF-*α* can promote IFN-*γ*-induced CD40 expression in MTHCs. In addition, only MTHCs cotreated with TNF-*α* and IFN-*γ* showed the profound ability to process the model antigen DQ-OVA. These data suggest that although both IL-4 and IFN-*γ* can play a role in progenitor cell development such as CD40 expression, IFN-*γ* has extra ability to cooperate with TNF-*α* to promote progenitor cells to differentiate into more biologically functional antigen-processing and antigen-presenting cells. These results have more clearly defined the biofunctional role of TNF-*α* and IFN-*γ* in APC development and maturation. 

Indeed, IFN-*γ* itself has been previously showed to be a strong CD40 inducer. Nguyen and Benveniste [30] and Nguyen et al. [31] have shown that IFN-*γ* strongly induces gene expression of CD40 and that IL-4 inhibits IFN-*γ*-induced CD40 expression through activation of STAT6 [30, 31]. Using a similar cell line MTHC-D2, Banyer et al. [23] showed that IFN-*γ* could induce the progenitor cells to express several genes important in DC development such as CD40 expression [23]. Our results, together with the above studies, suggest that IFN-*γ* itself has the ability to promote the development of APCs. In contrast, TNF-*α* alone or in combination with CD40 agonist is highly deficient, both physiologically and functionally in promoting DC maturation [32]. In the current study, we also showed that TNF-*α* alone could not increase CD40 expression of the progenitor cells. However, we demonstrate here that IFN-*γ* with an additional help of TNF-*α* can further promote CD40 expression and the development of the progenitor cells into functional APCs that can process an antigen and present a part of the antigen to the cell surface. A previous study showed that co-treatment of TNF-*α* and IFN-*γ* results in strong synergistic effects on the CD40 expression in activated HK-2 cells [33]. Although the cell type is different from ours, it supports our conclusion. 

The data in the current study provide a clear context for MTHCs to take up and process the model antigen DQ-Ovalbumin regulated by the cytokines. Antigen process is an important immunological step for antigen presentation to T lymphocytes and the subsequent immune response. The MTHCs had a weak capacity to process DQ-Ovalbumin in the presence of either GM-CSF or IL-4, but showed strong capacity in the presence of TNF-*α* and IFN-*γ*. TNF-*α* increased the ability of MTHCs treated with IFN-*γ*, but not with IL-4, to take up and process the antigen. In our study we showed that IL-4 plus INF-*γ* treatment was not better than INF-*γ* alone in DQ-OVA uptake and processing. This result is different from previous study by Banyer et al. [23] using MHTC-D2 cells where they showed that co-treatment with IL-4 and IFN-*γ* induced the greatest phagocytosis. This difference may be caused by different cell types used in the two studies or the difference in methodologies used in measuring cells to uptake and process antigens as DQ-OVA must be taken and processed by the cells to exhibit fluorescence signals. 

TNF-*α* and IFN-*γ* may simultaneously stimulate responsive genes in many cases [34, 35]. The increase may also be a result of crosstalk between the TNF-*α* and IFN-*γ* signalling pathways. It has been shown that TNF-*α* stimulation is able to induce transcription of IRF-1 and promotes IFN-*γ*-induced STAT1 activation [36, 37]. The synergistic effect of TNF-*α* and IFN-*γ* has been also shown to be mediated by NF-*κ*B pathway [38].

The MLRs can produce high levels of type-1 cytokines including IL-1, IL-6, IFN-*γ*, and TNF-*α*, but low or undetectable levels of type-2 cytokines such as IL-4 and IL-10 [39]. In our MLR experiments, the combined treatment with TNF-*α* and IFN-*γ* of the irradiated MTHCs showed a strong MLR response to allogenic T cells. This is consistent with a previous study on expression of class II antigens in human vascular smooth muscle cells (SMCs) [40]. IFN-*γ* is the only lymphokine secreted and capable of de novo induction of MHC class II antigen, HLA-DR expression in SMCs. TNF-*α* substantially enhanced IFN-*γ*-induced expression of HLA-DQ in MLR [40]. In macrophage, the ability of IFN-*γ* to synergize the effects of cytokines such as TNF-*α* and IL-4 is particularly important, as macrophages constantly receive multiple signals and need to integrate them to give a response appropriate to the extracellular milieu [38, 41]. Synergistic activation of the intercellular molecule 1 (ICAM-1) by TNF-*α* and IFN-*γ* is mediated by p65/p50 and p65/c-Rel and interferon-responsive factor Stat1 alpha (p91) that can be activated by both IFN-*γ* and IFN-*α* [42]. Both p50 and p65 proteins contribute to induction of complement factor B (Bf) gene by TNF-*α* and IFN-*γ* in macrophages [38]. 

IL-6, a proinflammatory cytokine, can increase adaptive immune response. It has also been shown to increase the maturation of DC [43]. We show that co-treatment of TNF-*α* and IFN-*γ* can increase the expression of IL-6 in MTHCs. IL-6 and TNF-*α* have been found to control N-glycosylation patterns of acute-phase plasma proteins [44]. Thus, there is a possible feed-forward regulation after IL-6 is stimulated by IFN-*γ* and TNF-*α*. Our data are consistent with a recent study that TNF-*α* induced mRNA and protein expression of IL-6 and MCP-1 in both fibroblasts from colorectal liver metastases and normal liver fibroblasts [45]. TNF-*α* synergized IFN-*γ* to upregulate expression of Bf gene in macrophages [38]. Huang et al. [38] identified that a region between −556 and −282 bp in the 5′ Bf promoter mediated TNF-*α* responsiveness and the synergistic effect of TNF-*α* and IFN-*γ* on Bf expression. Site-directed mutagenesis of an NF-kB-binding element in this region (−433 to −423 bp) abrogated TNF-*α* responsiveness and decreased the synergistic effect of TNF-*α* and IFN-*γ* on Bf expression [38]. It has been demonstrated that the interferon-stimulated response element and NF-kB mediate synergistic induction of murine IP-10 gene transcription by IFN-*γ* and TNF-*α* [46] to control IL-6 production [47]. This forward regulation can promote the rapid maturation of DCs. Btk-deficient DCs display a profound impairment of IL-6 and TNF-*α* production in response to stimulation by Toll-like receptor-8 (TLR-8) cognate agonist, ssRNA. Impaired TLR-8-mediated IL-6 and TNF-*α* production in antigen-presenting cells from patients with X-linked agammaglobulinemia was observed [48]. Here, we have not investigated the mechanisms that regulate the synergistic effects of TNF-*α* and IFN-*γ* on the upregulated expression of IL-6 in MTHCs, which warrants further study.

As a T-cell stimulating factor, IL-12 is involved in the differentiation of naïve T cells into Th0 cells which will further develop into either Th1 cells or Th2 cells. IL-12 can stimulate the growth and function of T cells and plays an important role in the activities of natural killer (NK) cells and T lymphocytes. IL-12 also mediates the enhancement of the cytotoxic activity of NK cells and CD8+ cytotoxic T lymphocytes [26]. It is well known that DCs express and secrete IL-12 [49]. CD1d-restricted T cells induced myeloid DCs to secrete IL-12 in the presence of a strong antigenic signal, and these DCs in turn activated naïve T cells to secrete Th1 cytokines [49]. T cells that produce IL-12 have a coreceptor, CD30, which is associated with IL-12 activity [26]. IL-1*β* alone significantly induced IL-12 production in DCs although TNF-*α* or IFN-*γ* induced modest levels of IL-12 production [50]. It appears that CD40 expression is negatively related to the IL-12 production in DCs. Murine bone-marrow-derived DCs incubated with live parasites or parasite extracts displayed enhanced levels of CD40, but no IL-10 or IL-12 could be detected in these DCs though small amounts of IL-6 and TNF-*α* were secreted by these DCs [51]. Similarly, we observed here that TNF-*α* reduced IFN-*γ*-induced production of IL-12 in MTHCs. But the mechanism of how this happens remains unclear though it is speculated that it could be related to TNF-*α* increased CD40 expression. However, further investigation is needed to confirm this. 

In summary, we show here that both IL-4 and IFN-*γ* can induce CD40 expression in MTHCs. TNF-*α* significantly increased CD40 expression induced by IFN-*γ*, but not by IL-4 in MTHCs. TNF-*α* synergized IFN-*γ* to take up and process antigen on cell surface and promoted IFN-*γ* to produce strong MLR response to allogenic T cells. TNF-*α* enhanced IFN-*γ*-induced expression of IL-6 mRNA and reduced the secretion of IL-12 induced by IFN-*γ*. The results indicate the key regulatory role of TNF-*α* in the development and differentiation of functional DCs from the MTHCs, which facilitate our understanding on DC development from hematological stem or progenitor cells.

## Figures and Tables

**Table 1:** Il-12 p40 protein in the cell culture medium measured by ELISA (pg/mL).

Treatment	0	1 : 1000 CD40L	1 : 500 CD40L
Control	<16	<16	43 ± 7
IFN	<16	34 ± 8	97 ± 6**
TNF+IFN	<16	30 ± 11	48 ± 6

*Notes*. ** *P* < 0.01 against both control and TNF-IFN. The values were mean ± SE of 3 experiments. The readings same as blank were regarded as no protein production and expressed as <16, the limit of the test.

## Abbreviations

MTHCs: Myb-transformed hematological cells
GM-CSF: granulocyte macrophage-colony stimulating factor
DCs: dendritic cells
TNF-*α*: tumour-necrosis factor-*α*
IFN-*γ*: interferon-*γ*
IL-4: interleukin-4
DQ-OVA: DQ-ovalbumin
FACS: fluorescence activated cell sorting
MLR: mixed lymphocyte reaction.
